# Nanoglasses: a new kind of noncrystalline materials

**DOI:** 10.3762/bjnano.4.61

**Published:** 2013-09-13

**Authors:** Herbert Gleiter

**Affiliations:** 1 Institute of Nanotechnology, Karlsruhe Institute of Technology, P.O. Box 3640, 76021 Karlsruhe, Germany and Nanjing University of Science and Technology, Herbert Gleiter Institute of Nanoscience, Building 340, Nanjing, Jiangsu 2 10094, P. R. China

**Keywords:** amorphous materials, ferromagnetism, nanoglasses, nanostructured materials, noncrystalline materials

## Abstract

Nanoglasses are a new class of noncrystalline solids. They differ from today’s glasses due to their microstructure that resembles the microstructure of polycrystals. They consist of regions with a melt-quenched glassy structure connected by interfacial regions, the structure of which is characterized (in comparison to the corresponding melt-quenched glass) by (1) a reduced (up to about 10%) density, (2) a reduced (up to about 20%) number of nearest-neighbor atoms and (3) a different electronic structure. Due to their new kind of atomic and electronic structure, the properties of nanoglasses may be modified by (1) controlling the size of the glassy regions (i.e., the volume fraction of the interfacial regions) and/or (2) by varying their chemical composition. Nanoglasses exhibit new properties, e.g., a Fe_90_Sc_10_ nanoglass is (at 300 K) a strong ferromagnet whereas the corresponding melt-quenched glass is paramagnetic. Moreover, nanoglasses were noted to be more ductile, more biocompatible, and catalytically more active than the corresponding melt-quenched glasses. Hence, this new class of noncrystalline materials may open the way to technologies utilizing the new properties.

## Review

### Introduction and basic concept

**Figure 1:**
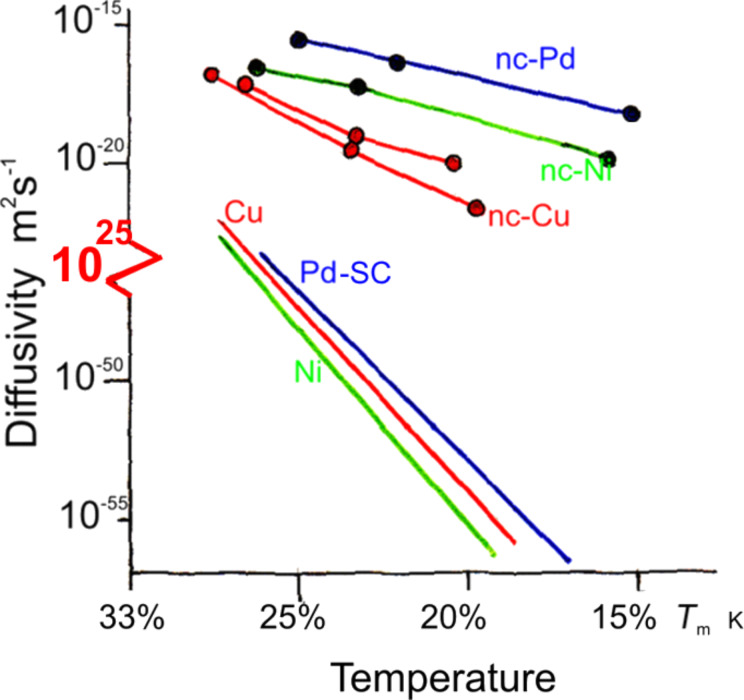
Comparison of the diffusivities in nanocrystalline (nc) Cu, Ni and Pd in comparison to the diffusivities in single crystals (SC) of Cu, Ni and Pd. *T*_m_ is the absolute melting temperature [1].

**Figure 2:**
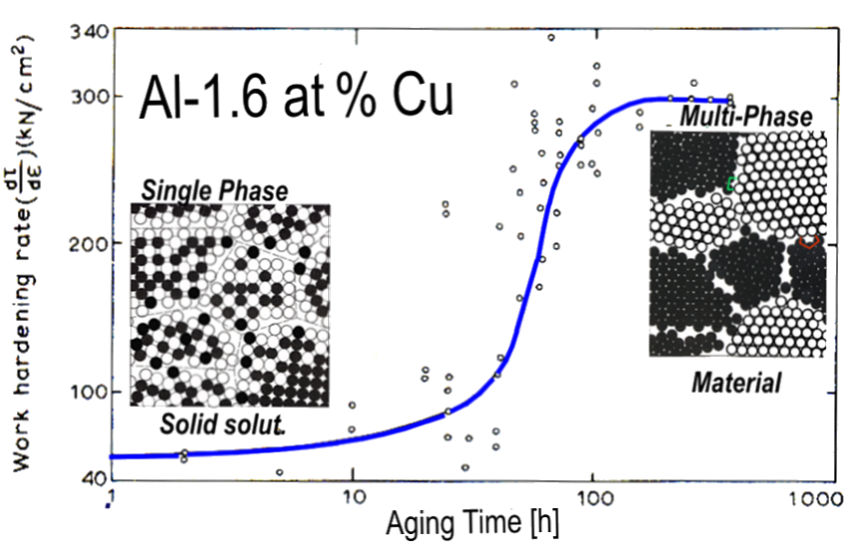
Work-hardening rate of (Al-1.6 at % Cu) crystals at room temperature after a solution treatment, water quenching, and aging at 190 °C for various times. The strain rate of the deformation process was 3 × 10^−4^ s^−1^. The aging at 190 °C results in a two-phase material consisting of precipitates embedded in a crystalline solid solution. Reprinted from [2] copyright (1963), with permission from Elsevier.

The majority of materials that have been used by mankind since the Neolithic age are crystalline materials. The oldest known examples are granite and quartz used for producing stone-age tools. More recent examples are light weight metals (e.g., Al), semiconductors (e.g., Si), materials with high strength (e.g., steels), superconductors, ferroelectrics, special ferromagnetic materials etc. The main reason for the preference of crystalline materials is the fact that one can control their properties by modifying their defect microstructures and/or their chemical microstructures. Figure 1 displays the remarkable enhancement of the diffusivities of Cu, Ni and Pd by varying the defect microstructure by means of introducing a high density of incoherent interfaces [1]. The modification of the properties of materials by varying their chemical microstructure is displayed in Figure 2 indicating the increase of the work hardening of an (Al-1.6 atom % Cu) alloy if the chemical microstructure (at constant chemical composition) is changed [2].

Glassy materials, although known for about 11000 years, have not yet been utilized to a similar extent. The main reason is that, so far, glasses are produced by quenching the melt and/or the vapor. Obviously, this approach does not permit the introduction of defect microstructures (e.g., similar to grain boundaries, Figure 1) or chemical microstructures (e.g., similar to the one shown in Figure 2). As a consequence, one cannot control the properties of today’s glasses by the controlled modification of their defect and/or chemical microstructures.

**Figure 3:**
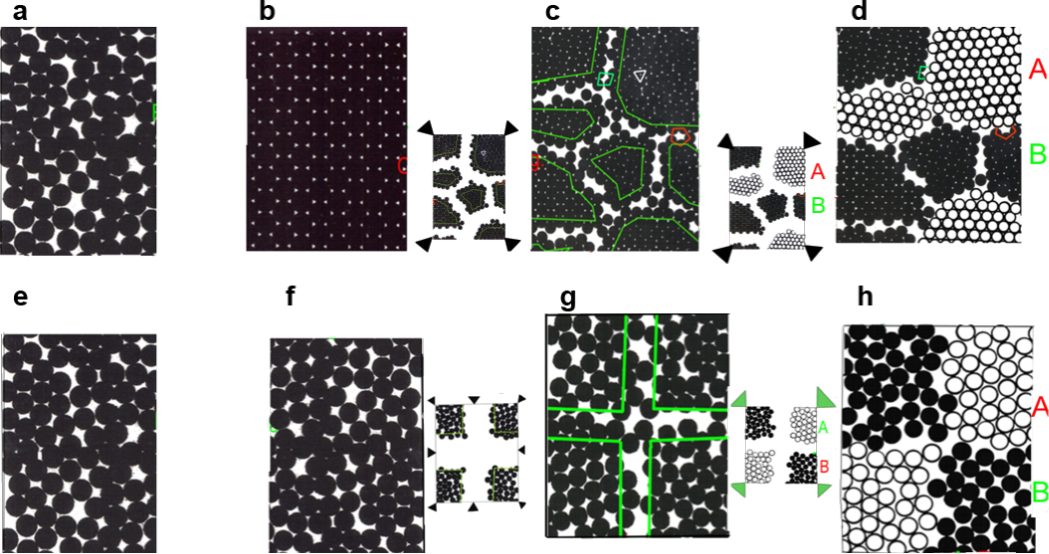
Figure showing the analogy between the defect and the chemical microstructures of nanocrystalline materials and nanoglasses. (a) Melt of identical atoms, (b) single crystal. The defect microstructure (c) and chemical microstructure (d) of nanocrystalline materials is compared with the corresponding defect microstructure (g) and the chemical microstructure (h) of nanoglasses. (f) displays the glassy structure obtained by quenching the melt shown in (e).

It is the idea of nanoglasses to generate a new kind of glass that will allow us to modify the defect and/or the chemical microstructures of glasses in a way comparable to the methods that are used today for crystalline materials. The basic concept of this approach is schematically explained by comparing the microstructures of nanoglasses and of nanocrystalline materials (Figure 3). If we consider a melt of identical atoms (Figure 3a and Figure 3e), we obtain a single crystal (Figure 3b) if we solidify this melt under conditions close to equilibrium. A nanocrystalline material with a high density of defects in the form of incoherent interfaces is obtained by consolidating nanometer-sized crystals (Figure 3c). If the consolidated nanometer-sized crystals have different chemical compositions, e.g., Ag crystals and Fe crystals (labeled as A and B in Figure 3d), we obtain a multiphase nanocrystalline material (Figure 3d).

The idea behind nanoglasses [3–6] is to apply an analogous approach, i.e., the consolidation of nanometer-sized glassy clusters in order to generate glasses with a high density of interfaces between adjacent glassy regions with either the same or with different chemical compositions. In other words, by consolidating nanometer-sized glassy clusters (Figure 3g), we generate a solid material that consists of nanometer-sized glassy regions (corresponding to the nanometer-sized crystallites in Figure 3c) connected by interfaces with an enhanced free volume due to the misfit between the atoms at the surfaces of adjacent glassy clusters. Due to the analogy of the nanometer-sized microstructures of both materials (Figure 3c and Figure 3g), the glass shown in Figure 3g is called a nanoglass. Again, if we consolidate nanometer-sized glassy clusters of different chemical compositions (Figure 3h), we obtain a multiphase nanoglass that is microstructurally analogous to the multiphase nanocrystalline material shown in Figure 3d. Hence, this kind of glass is called a multiphase nanoglass.

### Production of nanoglasses

So far, nanoglasses have been produced in the following three ways:

#### Inert-gas condensation

**Figure 4:**
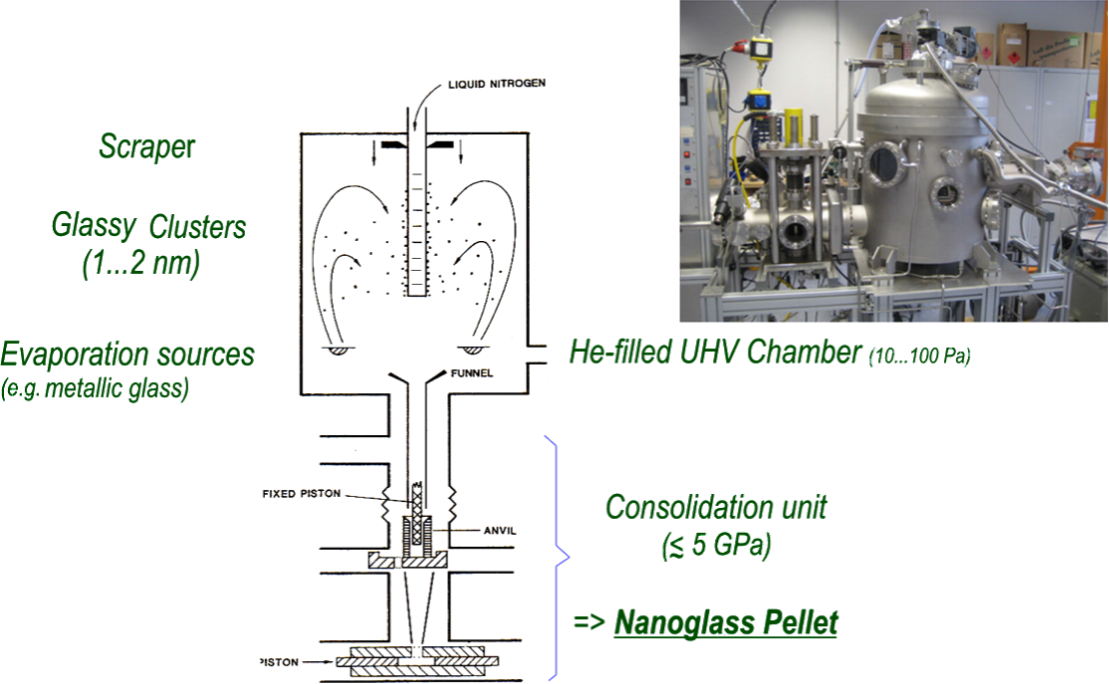
Production of nanoglasses by consolidation on nanometer-sized glassy clusters produced by inert-gas condensation [3–6]. Reprinted from [3] copyright (1989), with permission from Elsevier.

One way to produce nanoglasses is by means of inert-gas condensation (Figure 4). This production process involves the following two steps [3–6]. During the first step, nanometer-sized glassy clusters are generated by evaporating (or sputtering) the material in an inert gas atmosphere. The resulting clusters are subsequently consolidated at pressures of up to 5 GPa into a pellet-shaped nanoglass. So far, nanoglasses have been synthesized by inert gas condensation from a variety of alloys: Au–Si, Au–La, Cu–Sc, Fe–Sc, Fe–Si, La–Si, Pd–Si, Ni–Ti, Ni–Zr, Ti–P.

#### Magnetron sputtering

**Figure 5:**
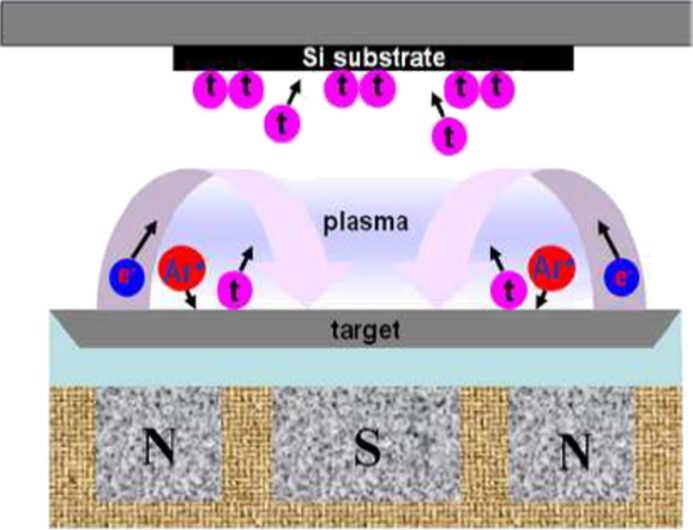
Synthesis of an Au-based nanoglass by magnetron sputtering. Reproduced with permission from [7].

This method (Figure 5) has been applied so far to Au-based metallic glasses [7–8]. The nanoglass obtained consisted of glassy regions with an average size of about 30 nm. Recent studies of the structure and the properties of nanoglasses produced by magnetron sputtering [7–8] suggest that their structure and properties are comparable to the ones of nanoglasses produced by inert gas condensation.

#### Severe plastic deformation

Due to the enhanced free volume in shear bands [9–10], the average free volume content of a glass was found to increase [10–11] with increasing plastic deformation. However, despite the similarity between the microstructural features of a nanoglass produced by consolidating nanometer-sized glassy spheres and a nanoglass produced by introducing a high density of shear bands, the results of recent studies by molecular dynamics (MD) [12–13] and Mössbauer spectroscopy of a ball-milled melt-quenched Fe_90_Sc_10_ glassy ribbon and a Fe_90_Sc_10_ nanoglass suggest that the atomic structure of both kinds of nanoglass differ. Moreover, the result obtained for an ionic material (LiAlSi2O_6_) suggests that the microstructure of the ball-milled LiAlSi_2_O_6_ glass is similar to the one of the nanocrystalline LiAlSi_2_O_6_ [14–16].

### Structural studies

#### Microscopy, positron annihilation spectroscopy (PAS) and X-ray diffraction

**Figure 6:**
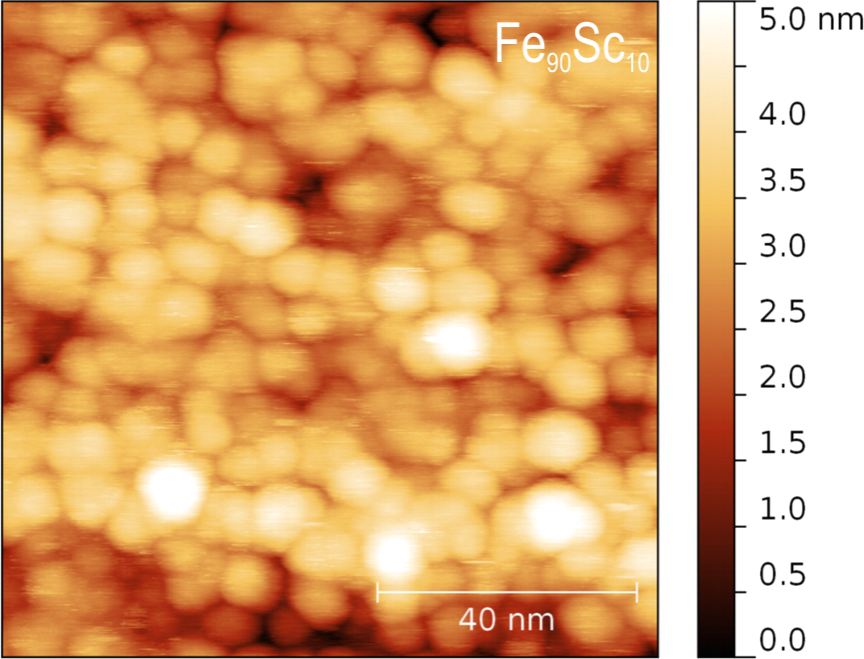
Constant-current scanning tunneling electron micrograph (STEM) of the polished surface of a Fe_90_Sc_10_ nanoglass specimen. The STEM reveals the granular structure of the Fe_90_Sc_10_ nanoglass produced by consolidating Fe_90_Sc_10_ glassy clusters with a pressure of 4.5 GPa [17].

The granular structure a of Fe_90_Sc_10_ nanoglass produced by consolidating Sc_75_Fe_25_ glassy clusters at a pressure of about 4.5 GPa is displayed (Figure 6) in the scanning tunneling microscopy image [17] of the polished surface of a nanoglass specimen.

**Figure 7:**
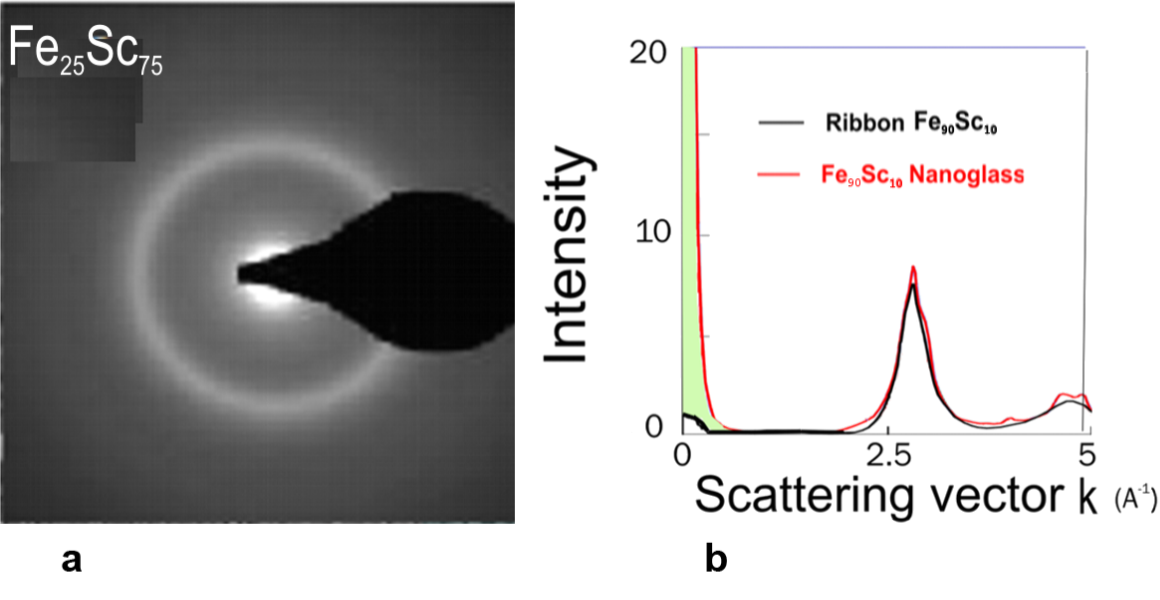
(a) Selected electron diffraction pattern of a Fe_25_Sc_75_ nanoglass. Reproduced with permission from [18]. (b) Wide-angle X-ray diffraction pattern of a Fe_90_Sc_10_ melt-spun ribbon and of a nanoglass with the same chemical composition.

**Figure 8:**
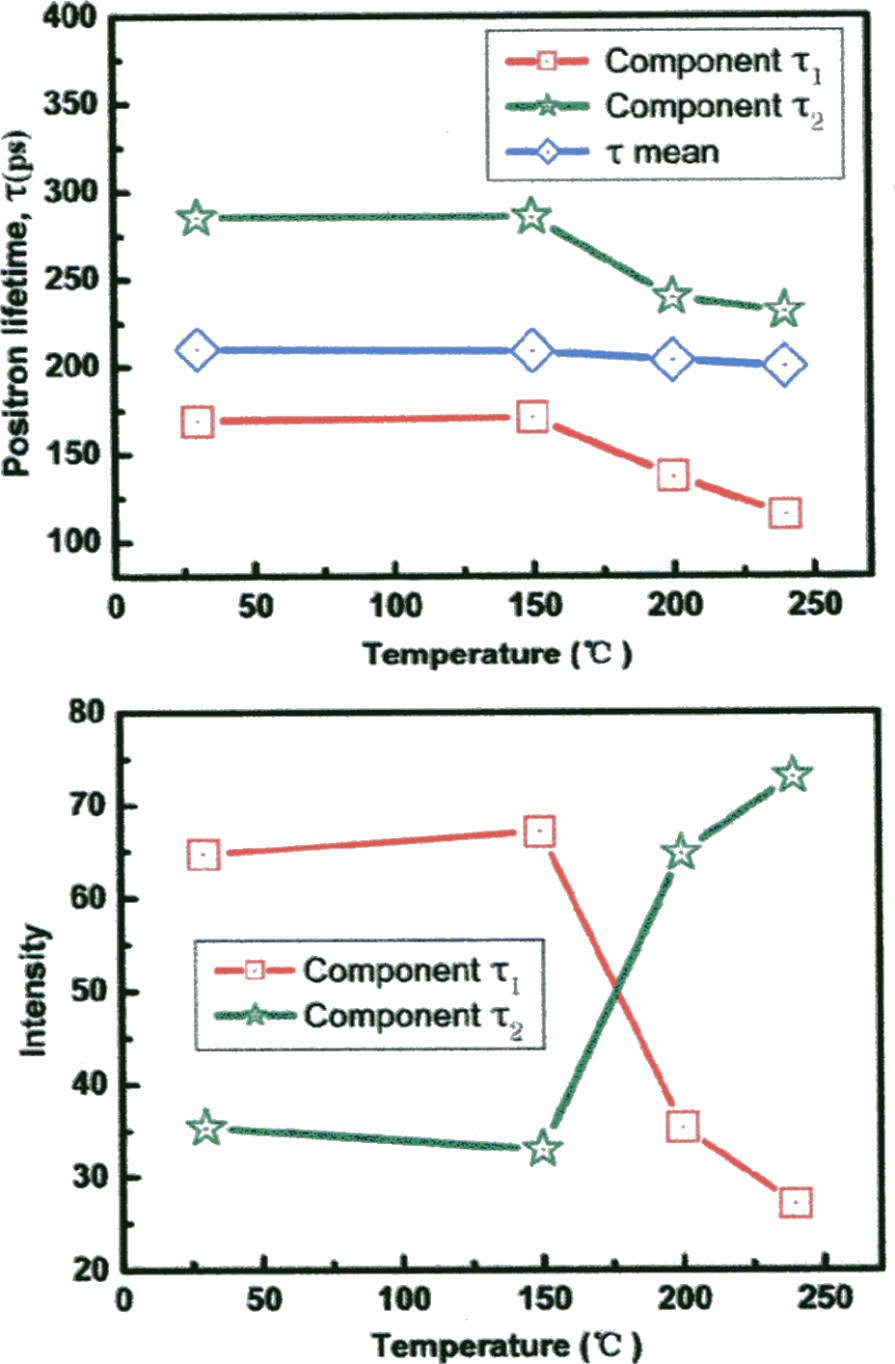
Upper figure: Positron lifetime of the components τ_1_ (red line), τ_2_ (green line) and the mean position lifetime τ_m_ (blue line) of a Sc_75_Fe_25_ nanoglass. Lower figure: Relative intensities of the same components (τ_1_ and τ_2_ in the upper figure) in the as-prepared state of the Fe_25_Sc_75_ nanoglass and during annealing of the same nanoglass. Reproduced with permission from [18].

The selected-area electron diffraction (SAED) pattern of the Fe_25_Sc_75_ nanoglass (Figure 7a) evidences [18] the amorphous structure. In fact, the wide-angle electron diffraction patterns of the nanoglass and of a melt-spun glassy ribbon were indistinguishable for large scattering vectors (Figure 7b). Positron annihilation spectroscopy (PAS) was applied (Figure 8) to examine the distribution of the free volume in as-prepared as well as in annealed Sc_75_Fe_25_ nanoglasses [18].

According to Figure 8, the following two lifetimes were observed: τ_1_ = 169 ps and τ_2_ = 285 ps [18]. The first (τ_1_ = 169 ps) compares well to the positron lifetime in the melt-spun glassy ribbons that have a similar chemical composition. Thus, this component is considered to originate from the interior of the consolidated glassy clusters. The second (τ_2_ = 285 ps) was seen exclusively in nanoglasses. Hence, it is supposed to originate from the glass–glass interfaces, which are characterized by an enhanced free volume. As seen in Figure 8 (lower figure), in the as-prepared Fe_25_Sc_75_ nanoglass the volume fraction of the glass–glass interfaces was about 65%. Upon annealing of the Fe_25_Sc_75_ nanoglass, the initial intensity of 65% of the τ_1_ component decreased to about 25% when the temperature was increased to 150 °C or above (Figure 8). At the same time, the τ_2_ component increased in intensity from 35 to about 75% (Figure 8). Positron lifetimes on the order of 350–500 ps, as indicators of nanovoids, were not observed.

**Figure 9:**
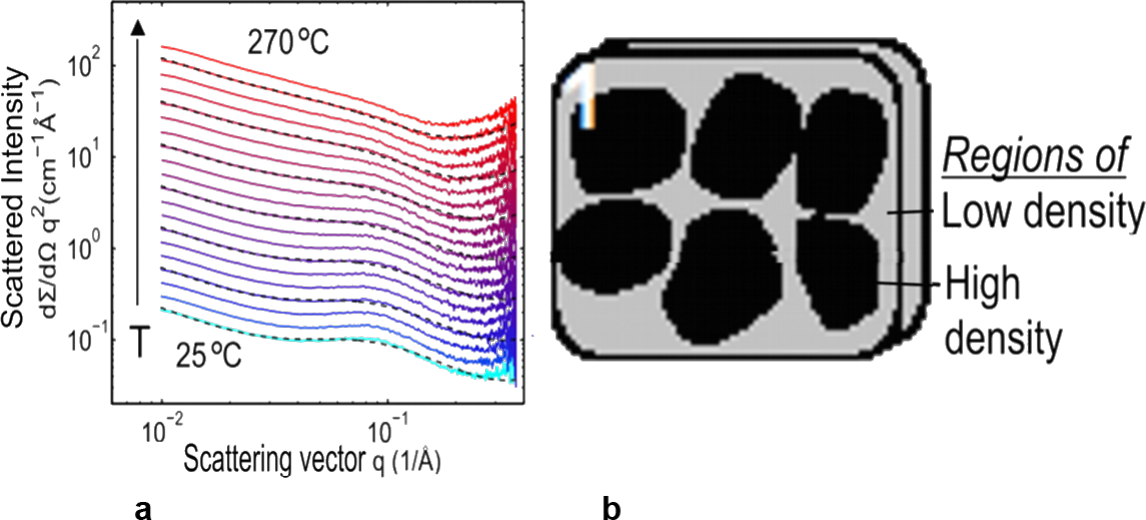
(a) q^2^-Weighted SAXS curves of a 4.5 GPa Fe_25_Sc_75_ nanoglass as a function of annealing temperature. The curves have been shifted vertically for clarity, except for the lowest curve. The scattering vector (q) is defined as 4π sin(θ)/λ, where θ is half of the scattering angle and λ is the wavelength. (b) Microstructural model of a nanoglass deduced by a Debye–Bueche transformation from the SAXS data shown in (a). The nanoglass consists of dense (nanometer-sized) regions (indicated in dark) embedded in a noncrystalline material with a lower density (gray regions). Reproduced with permission from [18].

In order to study the structure of nanoglasses, small-angle X-ray scattering experiments (SAXS) on Fe_25_Sc_75_ nanoglasses were carried out [18]. The SAXS curves obtained from these experiments (Figure 9a) are composed of a power-law component and a superimposed hump. The superimposed hump indicates that the structure of the nanoglass may be modeled (Figure 9b) as a two component system consisting of regions of high density and regions of lower density. By using the volume fractions of the glassy regions and of the glass–glass interfaces deduced from PAS (Figure 8), one obtains a difference in the electron density of about 17% between the density of the glass–glass interfaces and the density of the glassy regions. A fraction of this difference in electron density probably results from the different chemical compositions of the interfaces and the glassy regions. By elemental mapping, the interfaces of a Fe_25_Sc_75_ nanoglass were found to have a chemical composition of about Fe_15_Sc_85_. This Sc segregation to the interfaces contributes to the SAXS scattering intensity of the nanoglass. If this contribution is subtracted from the measured total SAXS scattering intensity, an excess free volume in the glass–glass interfaces of at least 6% was deduced (relative to the free volume in the adjacent glassy regions). This enhanced free volume in the glass–glass interfaces seems to agree with recent density measurements [19].

#### Electronic structure of nanoglasses

**Figure 10:**
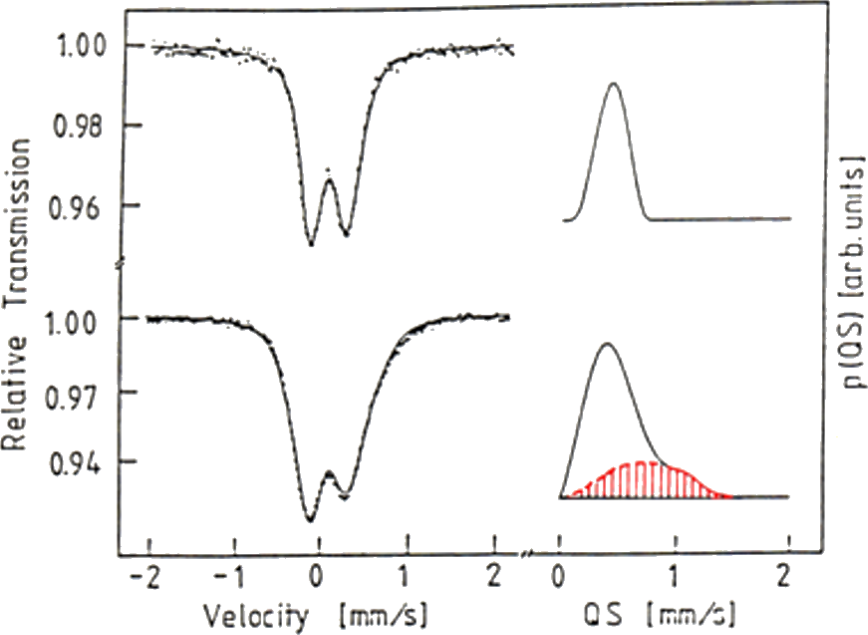
Comparison of the Mössbauer spectra and the corresponding quadrupole splitting (QS) distribution (*p*(QS)) of a melt-spun Pd_72_Fe_10_Si_18_ metallic glass (upper part of the figure) and of a nanoglass with the same chemical composition generated by consolidating 3.6 nm sized glassy spheres. Reprinted from [3] copyright (1989), with permission from Elsevier.

The different atomic arrangements in the glass–glass interfaces and in the adjacent glassy regions as well as interfacial segregation effects seem to result in different electronic structures in both regions. A first indication of the different electronic structure was the observation [3] that the Mössbauer isomer shift (IS) of the interfacial component of PdSiFe glasses (Figure 10) was larger than the IS value of the melt-cooled glass.

**Figure 11:**
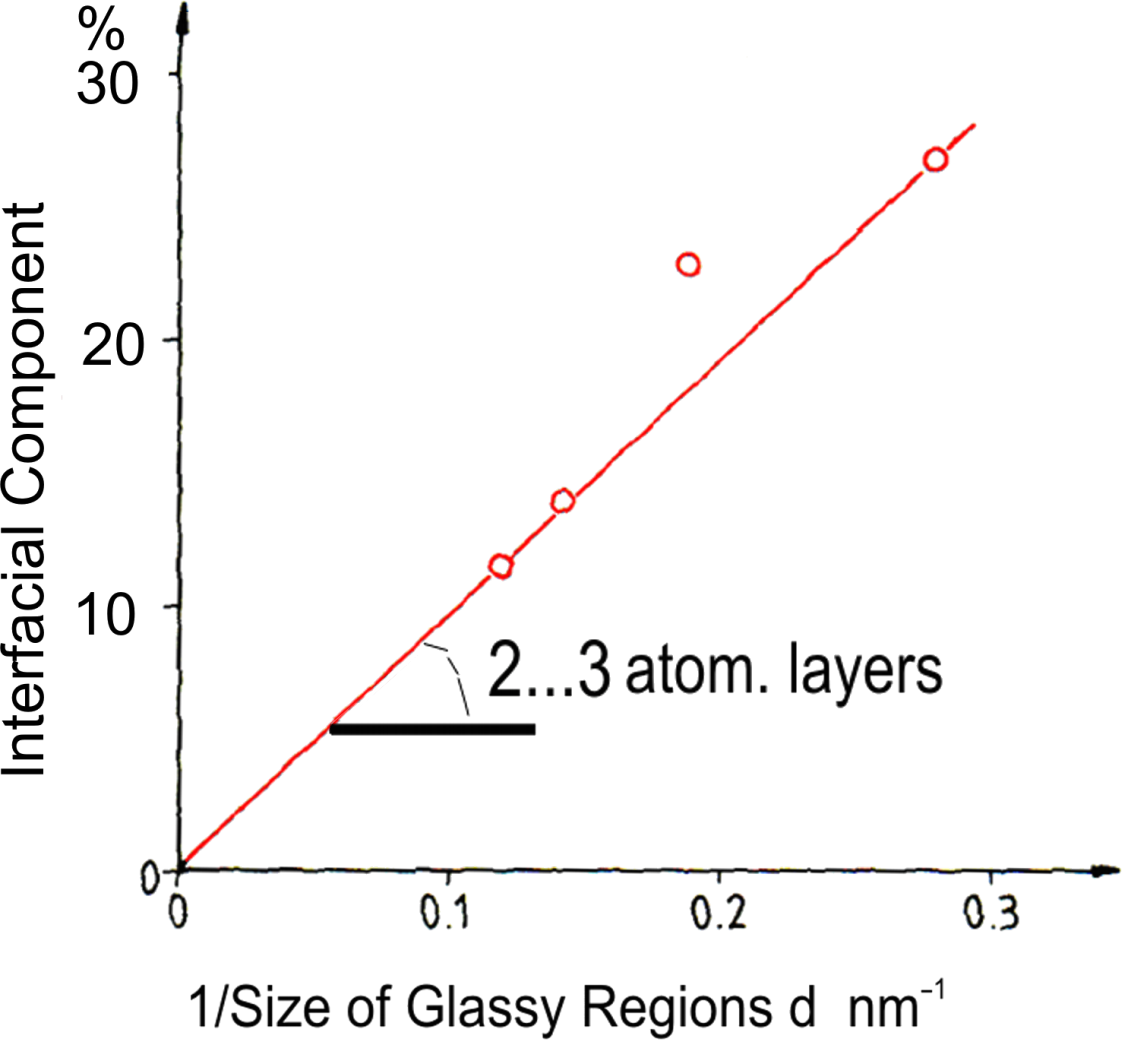
Relative spectral fraction of the interfacial component versus the inverse size of the glassy regions [3] of the nanoglass shown in Figure 10. The slope of the curve indicates a width of the boundaries between the glassy regions of the nanoglass of about 2 to 3 atomic layers. Reprinted from [3] copyright (1989), with permission from Elsevier.

Figure 10 displays the Mössbauer spectra and the respective quadrupole splitting (QS, right side of the figure) distributions of a melt-spun Pd_72_Fe_10_Si_18_ glass and a nanoglass with identical chemical compositions [3]. As may be seen the QS distribution of the nanoglass consists of the following two components. One component coincides with the peak of the melt-spun glass, and a second one (at about 0.9 mm·s^−1^, indicated in red in Figure 10) was observed in the nanoglass only suggesting that it originates from the interfaces between the glassy regions. This interpretation agrees with the observation [3] that the area under the second peak scales approximately with the volume fraction of the interfaces in the nanoglass (Figure 11).

**Figure 12:**
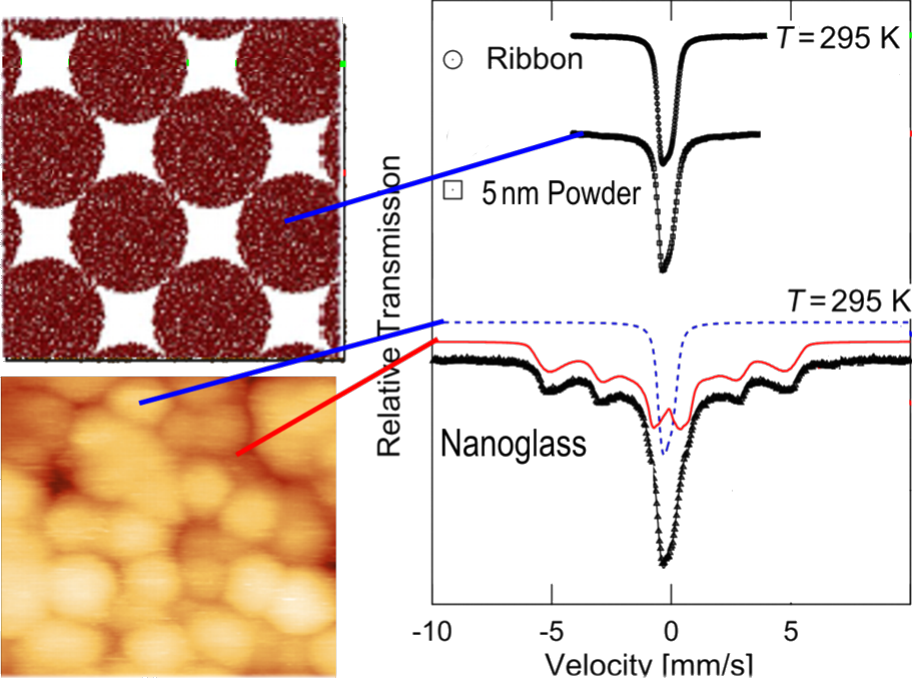
Mössbauer spectra recorded at 295 K for the melt-spun ribbon, the nanosphere powder prior to consolidation and the nanoglass. The diameter of the consolidated glassy clusters (visible in the micrograph displayed on the lower left) was 8 nm (cf. Figure 6). In all cases the chemical composition was Fe_90_Sc_10_. The melt-spun ribbon and the unconsolidated nanosphere powder exhibit identical single line spectra typical for paramagnetic materials (upper right side). The nanoglass spectrum (lower right side) may be separated into a paramagnetic component (PM, blue), with a spectral shape similar to the ribbon (or the powder) and a ferromagnetic (FM, red) component. This component consists of six lines that are a characteristic feature of ferromagnetic materials. The straight red and blue lines indicate the suggested location of the two different components (FM and PM) of the Mössbauer spectrum within the structure of the nanoglass [17].

From the slope of the line shown in Figure 11, the thickness of the interfaces was deduced to be 0.4 nm, corresponding to about two to three atomic layers. Hence, the structural model (Figure 3g) of a nanoglass (consisting of nanometer-sized glassy regions connected by glass–glass interfaces with a reduced density) seems to agree with the results reported above by using Mössbauer spectroscopy (Figure 10) as well as with the SAXS results (Figure 9). A further observation indicating the different electronic structure of the glassy and of the interfacial regions was reported [17] for Fe_90_Sc_10_ glasses. Melt-cooled glassy ribbons of Fe_90_Sc_10_ glasses and a nanoglass with the same chemical composition displayed different Mössbauer spectra (Figure 12).

The single line spectra of the ribbon as well as of the isolated nanometer-sized glassy spheres indicate that both are paramagnetic. The spectrum of the consolidated spheres (Figure 12 lower-right side) consists of the following two components: (1) a paramagnetic component (indicated in blue in Figure 12) similar to the spectrum of the ribbon or of the isolated Fe_90_Sc_10_ nanometer-sized clusters and (2) a ferromagnetic component (six-line subspectrum: red curve in Figure 12).

**Figure 13:**
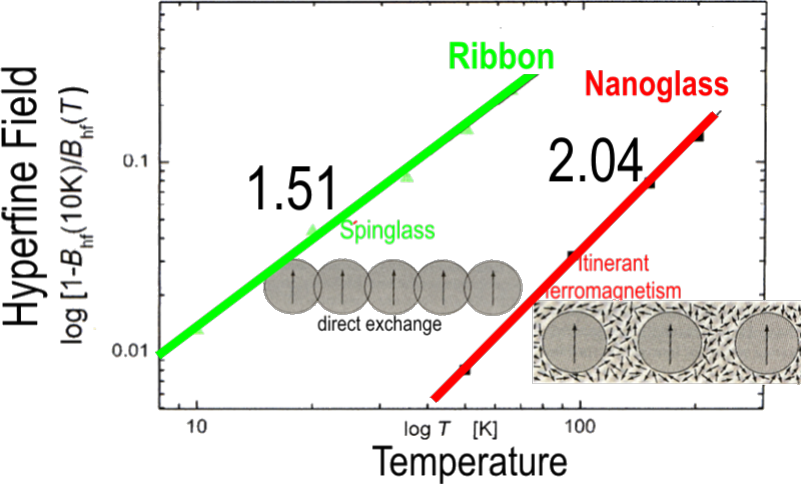
Diagram displaying the temperature dependence of the measured magnetic hyperfine field (Bhf) of a melt-spun ribbon and a nanoglass with the same chemical composition (Fe_90_Sc_10_). The *T*^1.51^ dependence observed for the melt-spun ribbon agrees with spin wave theory [21]. In the case of the Fe_90_Sc_10_ nanoglass a *T*^2.04^ temperature dependence was observed, which evidences itinerant ferromagnetism in the nanoglass. Magnetic Compton scattering experiments performed by using the same specimens indicate that the itinerant ferromagnetism in the nanoglass is caused by negative spin polarized sp-like itinerant electrons [21–24].

As the ferromagnetism at ambient temperature is observed only if the Fe_90_Sc_10_ nanospheres are compacted (Figure 12), one is led to conclude that it is the regions between the spheres that are magnetically ordered. Ferromagnetism has never been observed in melt-spun or vapor-deposited amorphous Fe*_x_*Sc_100−_*_x_* alloys at ambient temperatures (irrespective of the chemical composition). In other words, neither interfacial segregation nor inhomogeneous elemental distributions can account for the ferromagnetism observed in the Fe_90_Sc_10_ nanoglass. In Figure 13 the temperature dependence of the magnetic hyperfine field of the melt-quenched ribbon of a Fe_90_Sc_10_ glass is compared with the one of the interfacial regions of a Fe_90_Sc_10_ nanoglass [20]. As may be seen, the ribbon exhibits the typical spin glass behavior based on the polarization and coupling of localized 3d electrons. This behavior is characterized by a slope of 1.5 in the magnetic hyperfine field (*B*_hf_) versus temperature (*T*) plot as displayed in Figure 13.

Clearly, this result deviates from the behavior of the interfacial component of the nanoglass with the same chemical composition. In the case of the nanoglass, the slope of the *B*_hf_ versus *T* plot is found to be about 2 (Figure 13). This slope indicates [21–24] a dominant contribution of the itinerant electrons to the magnetic coupling in the nanoglass interfaces. The same conclusion is suggested by the magnetic Compton profile of the chemically identical nanoglass [21] indicating that the itinerant ferromagnetism of the Fe_90_Sc_10_ nanoglass is based on spin-polarized sp-like itinerant electrons [21–24].

If the Young’s moduli of Sc_75_Fe_25_ nanoglasses [25–26] and Au_52_Ag_5_Pd_2_Cu_23_Si_10_Al_6_ nano-glasses [7] are compared with the Young’s moduli of the corresponding melt-quenched glassy ribbons, the moduli of the nanoglasses are found to be higher. As the atomic density in the nanoglass interfaces is reduced in comparison to a melt-quenched glass with the same chemical composition (Figure 9), the enhancement of the Young’s moduli indicates that the interatomic interaction, i.e., the electronic structure of the nanoglass differs from the one of the corresponding melt-quenched glass. This conclusion agrees with the results of recent nuclear resonant vibrational spectroscopy (NRVS) measurements performed at 300 K. The mean interatomic force constant (*P*) in a melt-spun Fe_90_Sc_10_ was 138.195 N/m whereas the one in the nanoglass was almost 10% higher (147.965 N/m) [20]. It should be noted that the Young’s modulus measurements for the nanoglass exhibited a significantly larger scattering than the ones for the ribbon. One reason may be that the nanoglass has a larger porosity or local fluctuations in the chemical compositions. However, the scattering was in all measurements less than the enhancements of the moduli. Moreover, the NRVS data do not depend on porosity.

#### Structural stability

**Figure 14:**
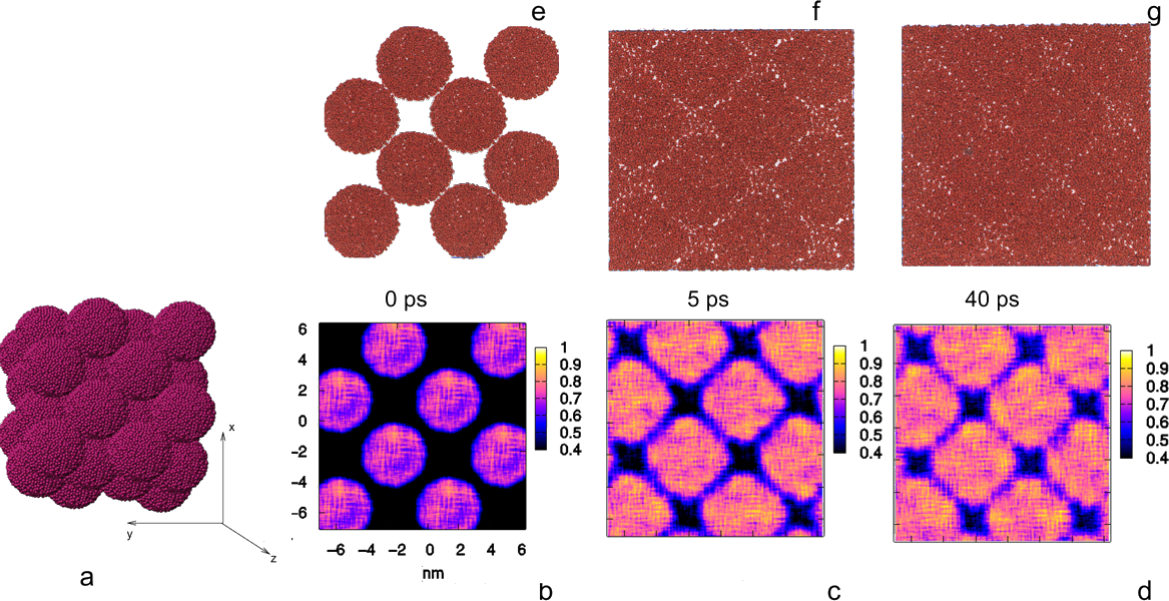
Molecular dynamics simulation of the consolidation of a nanoglass at 300 K [27]. The nanoglass is obtained by sintering nanometer-sized (5 nm diameter) glassy spheres (of Ge) at a pressure of 5 GPa. (e–g) displays the atomic structure of the nanoglass by showing the position of the Ge atoms within a thin slab of material cut out (parallel to the *x*–*y*-plane, (a)) of the block nanoglass. The density distribution in the nanoglass block is shown in (c,d). The contour plots display the atomic density relative to the bulk value (cf. the density scales on the right side of (b–d)). As the sintering process proceeds (b–d), the density in the glass–glass interfaces increases and the interfaces and interface junctions become wider [27]. Reproduced with permission.

Due to the important role of the structural stability of nanoglasses, this issue is addressed in Figure 14 on application of molecular dynamics (MD).

Although already discussed in [28], the essential results obtained so far are briefly repeated here in order to provide a complete picture of our present understanding. Figure 14a–g compares the results of a molecular dynamics (MD) simulation of the microstructural evolution of a three-dimensional nanoglass formed by sintering glassy spheres of Ge [27]. The sintering process occurred under a hydrostatic pressure of 50 kbar at 300 K. All Ge spheres were of the same size (diameter 5 nm) and were (Figure 14a) arranged in an initial face-centered cubic (fcc) structure. Figure 14b–d and Figure 14e–g present the computed evolution of the atomic structure of this Ge nanoglass. The figures in the upper row of Figure 14 (Figure 14e–g) show the arrangement of the atoms in a thin slab (Figure 14a) that was cut out of the sintered Ge nanoglass. The fcc arrangement of the glassy Ge spheres (Figure 14a) initially results in a regular arrangement of voids between these spheres (Figure 14e). On continuation of the sintering process (Figure 14f,g), the size (volume) of the voids between the spheres reduces in size, and in the contact regions between adjacent spheres glass–glass interfaces and junctions of several interfaces are formed. As the sintering process proceeds, these interfacial regions of enhanced free volume increase in width, i.e., the fluctuations of the free volumes delocalize (Figure 14c,d).

If the results of the experimental observations (Figure 8 and Figure 9a) are compared with the MD results obtained for Ge (Figure 14), the following discrepancy is apparent. The MD results suggest that in the nanoglasses the delocalization process of the interfaces and junctions, even at relatively low temperatures (e.g., at 100 K), occurs within a few nano- or picoseconds. However, the experimental observations (Figure 8 and Figure 9a) suggest the delocalization to require, even at 450 K (Figure 8), hours or more. This discrepancy may result from one or both of the following reasons. The first reason may be a chemically inhomogeneous microstructure of the nanoglass as was reported [18] for the as-consolidated Sc_75_Fe_25_ nanoglasses. In fact, the measurements indicated an enhanced Sc concentration at the interfaces of the FeSc nanoglass. This enhanced concentration may delay the interfacial delocalization. For example, in the case of Y–Fe nanocrystalline materials grain growth was found to cease due to the solute segregation to the interfaces [29–31] (“solute drag effect”) [32–34]. In principle, the same effects are likely to apply to the structural changes of nanoglasses. Moreover, the solute segregation at the glass–glass interfaces will change (cf. the previous section on the electronic structure of nanoglasses) the electronic structure of the nanoglasses at the interfaces and hence the interatomic potentials at the interfaces. However, in all MD simulations of the delocalization, the interatomic potential was assumed to be the same everywhere in the nanoglasses.

In fact, the significance of electronic effects for the stability of metallic clusters is well known [35]. For example, if metallic clusters are prepared from the vapor phase, certain cluster sizes (called “magic clusters”) are known to be more stable than other sizes due to their low energies. Comparable effects have been reported for polymer glasses as well as glasses made up by individual atoms or small molecules [36–37]. In fact, by means of a combination of experimental and computational techniques Sheng et al. [38] have analyzed the atomic-level structure of amorphous alloys in terms of geometrically distinct polyhedron types that appeared with high frequencies. All of these polyhedra were found to be Frank–Kasper polyhedra that involve the minimum number of disclinations. Moreover, as already pointed out by Bernal [39], the packing of molecular clusters leaves behind a pattern of cavities (“canonical holes”) in the resulting glassy structure. The size and distribution of these cavities has been shown to be important for the stability and enthalpy of glasses. In fact, such a process has been shown to lead to a “structural arrest” [40] if the energy reduction due to the interfacial delocalization is less than the energy required to replace the relaxed stable structure of the glassy clusters by a structure of lower stability and higher free energy.

#### Structural model of nanoglasses

**Figure 15:**
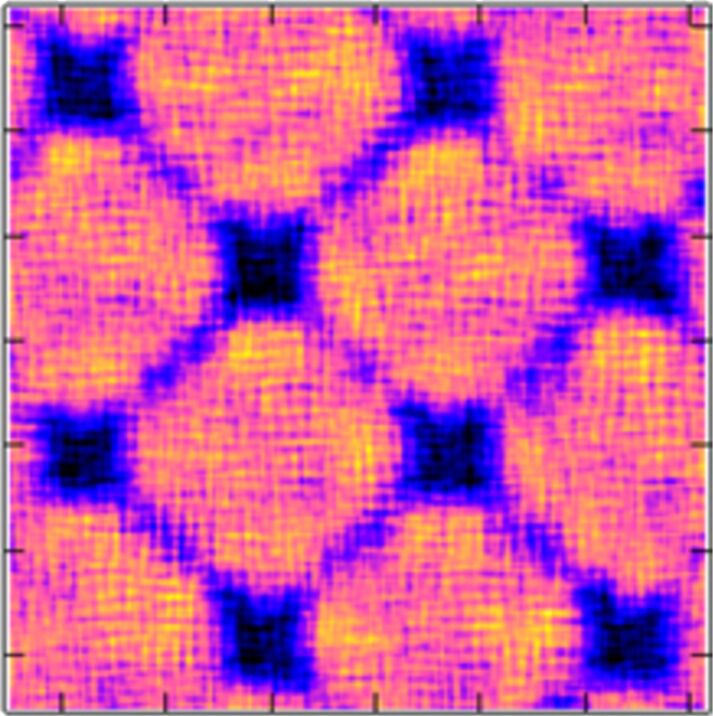
Proposed model of the structure of a nanoglass [27]. Reproduced with permission. According to the results reported in this paper, nanoglasses consist of two kinds of noncrystalline regions: First, regions that have the atomic structure of glasses produced by quenching the melt. These regions (red–yellow color) result from the consolidated nanometer-sized glassy clusters. The second structural component of nanoglasses (indicated in blue and black) has a new kind of noncrystalline structure. This new structure is, according to the results reported, characterized (relative to the glassy structure of the chemically identical material indicated in the red–yellow regions) by a new atomic as well as a new electronic structure. The new atomic structure is characterized by a reduced density, an enhanced spacing between next-nearest-neighbor atoms and a reduced number of nearest-neighbor atoms. The new electronic structure is suggested by the observation of a reduced s-electron density (Mössbauer spectroscopy, Figure 10), an enhanced Young’s modulus, an atomic force constant in NRVS, an enhanced Curie temperature and enhanced hyperfine field (Figure 12) as well as itinerant ferromagnetism instead of a spin-glass structure (Figure 13).

In summary the structural model of metallic nanoglasses that emerges from these observations is as follows (Figure 15). Nanoglasses are noncrystalline solids consisting of the following two regions. There are regions (red and yellow in Figure 15) with the same atomic structure as a glass produced by quenching the melt. These regions originate from the nanometer-sized glassy spheres that were consolidated in order to produce the nanoglass. Between these glassy regions, interfacial regions (dark blue in Figure 15) exist. In these interfacial regions, layers of a new kind of noncrystalline atomic structure (different from the atomic structure in the red and yellow regions) are formed. This new noncrystalline structure is associated with an electronic structure that differs from the one of the corresponding melt-quenched glass. The new kind of noncrystalline structure is, according to the results reported above, characterized (relative to the glassy structure in the red yellow regions) by a reduced density, an enhanced spacing between next-nearest-neighbor atoms and a reduced number of nearest-neighbor atoms. The new electronic structure of these interfaces is suggested by the observation of a reduce s-electron density (Mössbauer spectroscopy), an enhanced Young’s modulus and atomic force constant in NRVS, an enhanced Curie temperature and enhanced hyperfine field as well as itinerant ferromagnetism instead of a spin glass structure. In other words, nanoglasses seem to consist of the following two noncrystalline phases: one phase with a glassy structure and another phase with a new kind of noncrystalline atomic structure as well as a new electronic structure.

### Properties of nanoglasses

#### Ferromagnetism in FeSc nanoglasses

**Figure 16:**
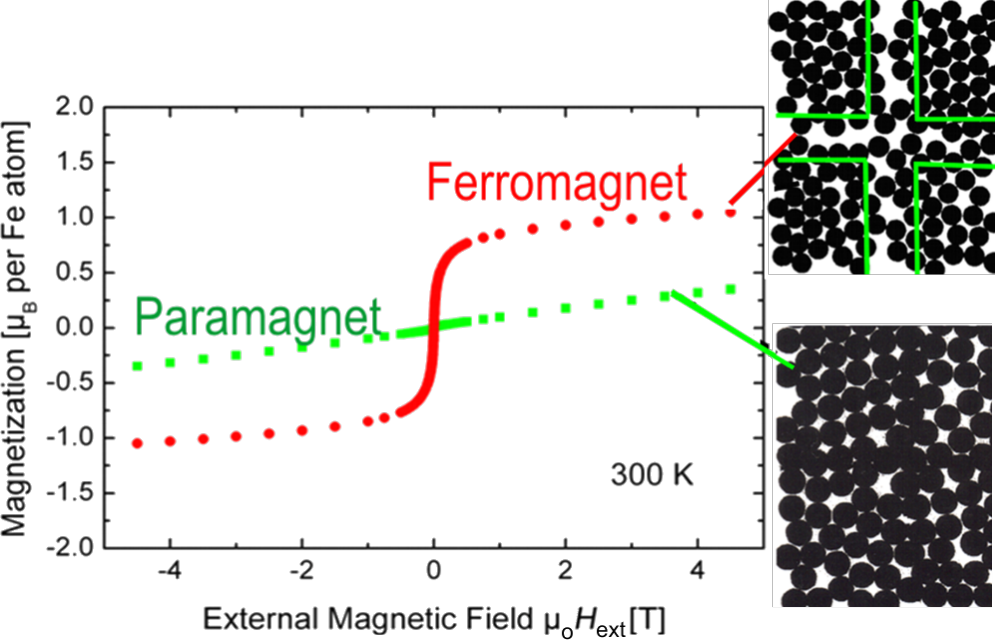
Magnetization curves (magnetization versus external magnetic field) of a nanoglass sample (red) and a melt-spun ribbon (green) at 300 K. The ribbon exhibits paramagnetic behavior, while the nanoglass shows a curve characteristic for ferromagnetic materials with a magnetization of 1 μ_B_ per Fe atom in the applied magnetic field of 4.5 T. The error bars are smaller than the symbols [17]. The chemical composition of both specimens was Fe_90_Sc_10_.

Figure 16 presents the magnetization curves, *M* (magnetization) versus *H* (external magnetic field), of a nanoglass sample and of a melt-spun ribbon having the same chemical composition (Fe_90_Sc_10_) [17]. The *M*-versus-*H* loop, recorded at ambient temperature, evidences that the ribbon is paramagnetic at this temperature, in agreement with the results reported in the literature [41–42]. In contrast, the magnetization curve of the Fe_90_Sc_10_ nanoglass indicates that it is ferromagnetic and it exhibits an average magnetization of about 1.05 μB per Fe atom. According to the Mössbauer spectrum of the nanoglass (Figure 12) the ferromagnetism is associated with the glass–glass interfaces. The results of the Mössbauer spectroscopy (Figure 12) seem to rule out crystallites of bcc-Fe or Fe-oxide crystallites as the origin of the ferromagnetism. Only small amounts (<10%) of nanometer-sized bcc-Fe crystallites were revealed in the low temperature Mössbauer spectra [17]. These crystallites are superparamagnetic at ambient temperature.

#### Plastic deformation of nanoglasses

**Figure 17:**
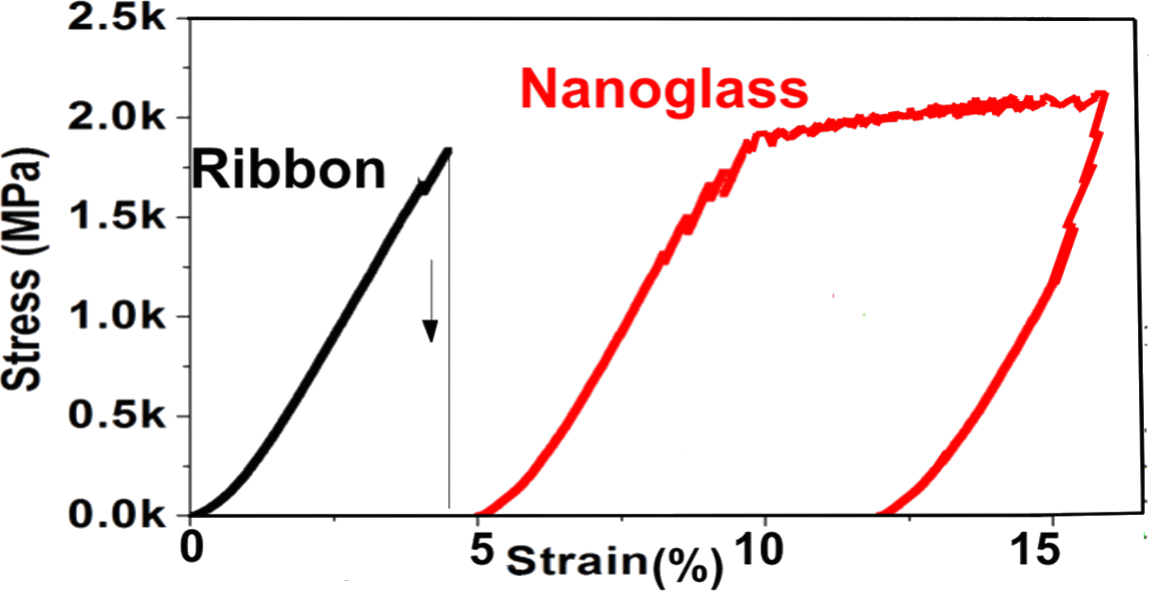
Stress–strain curve of a Sc_75_Fe_25_ nanoglass and of a melt-spun ribbon with the same chemical composition [43].

**Experimental observations:** By using microcompression experiments [43], the deformation behaviors of the following two kinds of glasses were investigated: (1) a melt-quenched ribbon of a Sc_75_Fe_25_ metallic glass, and (2) an as-prepared Sc_75_Fe_25_ nanoglass. The stress–strain plots of these two glasses are displayed in Figure 17.

As may be seen, the glassy ribbon exhibits brittle fracture at a strain of around 5% and stresses between about 1900 and 2200 MPa with a plastic deformation of less than 1%. The behavior of the glassy ribbon differed from the plastic deformation observed for the corresponding Sc_75_Fe_25_ nanoglass, as shown in Figure 17. The Sc_75_Fe_25_ nanoglass (in the as prepared state) was found to yield at a stress of about 1250 MPa. Beyond the yield point, the nano-glass exhibited extensive plastic flow of up to about 15%. The fracture stress was about 1950 MPa, which is comparable to the fracture stress of the ribbon. These results were interpreted in terms of the different microstructures of the melt-quenched ribbon and the nanoglass. In structurally homogenous ribbons, only one (or a few) shear bands are known to be nucleated under sufficiently high applied stresses. Plastic flow is limited to these shear bands and frequently results in fracture after an overall plastic deformation of less than 1%. However, in the nanoglass, plastic flow was noted to occur rather homogenously throughout the entire volume. By analogy with the deformation of single crystalline and polycrystalline materials, different plastic deformation modes of the ribbon and of the nanoglass were suggested [42–43] to result from numerous intersecting multiple shear bands that nucleate at the glass–glass interfaces of the nanoglass. In fact, along the tensile direction, the nanoglass clusters were noted to be stretched and a considerable fraction of the plasticity seems to originate from the elongation of these clusters [8]. The interpretation [42–43] of the different plastic deformation modes of the ribbon and of the nanoglass as resulting from numerous intersecting multiple shear bands that nucleate at the glass/glass interfaces of the nanoglass seems to agree with the following observations on the enhanced plasticity of glasses. Lee et al. [44] obtained enhanced plasticity in ZrCuNiAl glasses with a heterogeneous microstructure consisting of hard regions surrounded by soft ones. Other approaches, such as cold rolling [44], elastostatic compression [45] or nanometer-sized structural heterogeneities [46–47] have also been shown to result in enhanced plasticity.

The work hardening of nanoglasses noted in the experiments mentioned above [43] seems also to be related to the numerous intersecting shear bands. This interpretation agrees with the observations of Cao et al. [48]. In fact, enhanced plasticity, work hardening and high fracture stresses were also noted if numerous shear bands had been introduced in metallic glasses by cold rolling prior to the deformation tests. Similarly, Takayama [49] observed work-hardening phenomena in highly drawn metallic glass wires, and attributed this behavior to the intersection of shear bands. Moreover, work hardening of glasses has also been reported for glassy composites with reinforcing crystalline phase [50] and for glasses containing microstructural heterogeneities [46].

**Figure 18:**
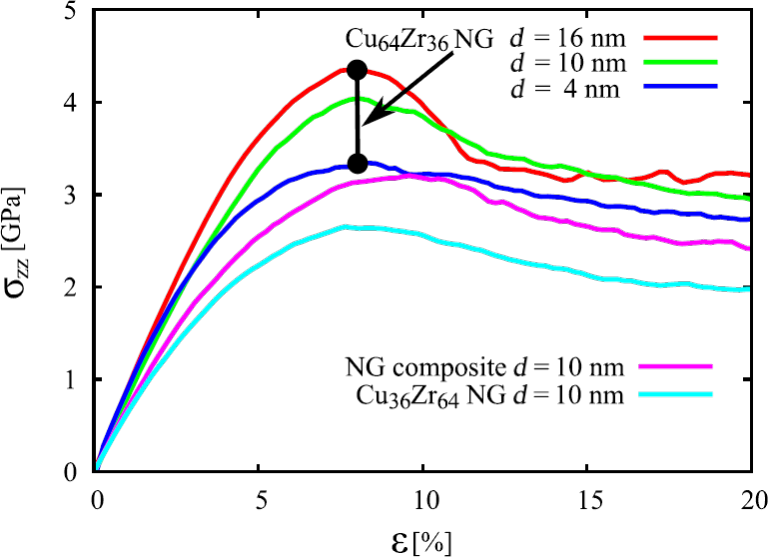
Calculated stress–strain curves for Cu_64_Zr_36_ nanoglasses with glassy regions with diameters of 4, 10 and 16 nm. These curves are shown together with the result obtained for a layered nanoglass composite consisting of layers alternating between Cu_64_Zr_36_ and Cu_36_Zr_64_ [51].

**MD simulations:** Recent studies [51–52] of the mechanical properties of a Cu_64_Zr_36_ nanoglass and of a bulk metallic glass with the same chemical composition under tensile load by means of molecular dynamics support the ideas proposed above. In fact, the following two types of nanoglasses were studied [51–52]. One nanoglass was chemically homogeneous. In the second nanoglass, Cu-atoms were segregated to the interfaces between the glassy regions. Both glasses were deformed at 50 K with a constant strain rate of 4 × 10^7^ s^−1^. The stress–strain curves for both nanoglasses are displayed in Figure 18.

**Figure 19:**
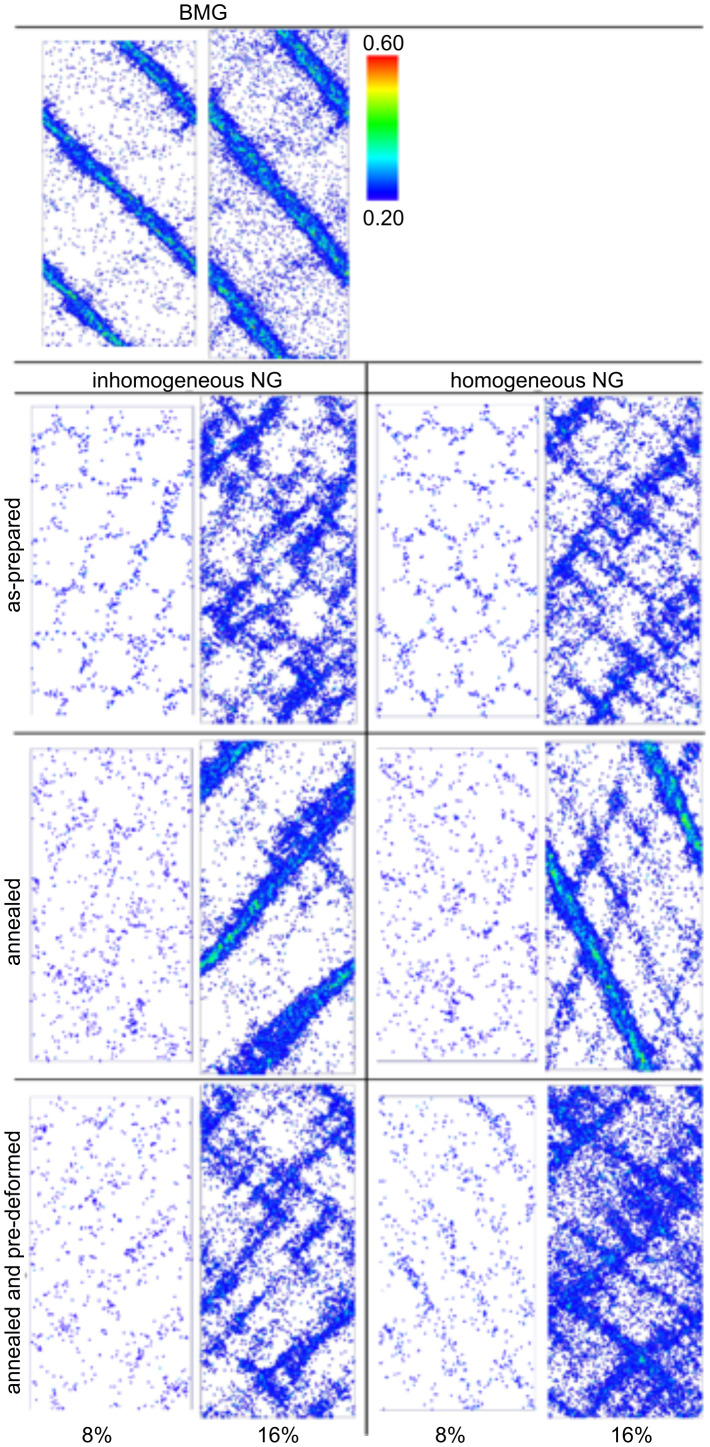
Local atomic shear strain for chemically inhomogeneous (Cu-enriched interfaces) and chemically homogenous nanoglasses in the as prepared, the annealed, and the pre-deformed state. Reproduced with permission from [52].

**Figure 20:**
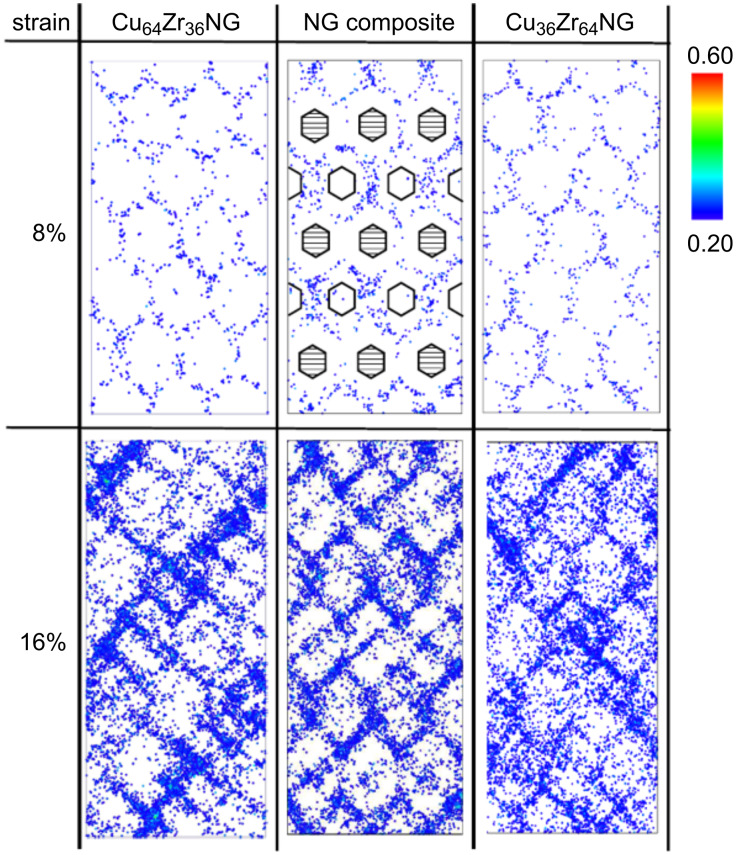
Left: Atomic shear strain in Cu_64_Zr_36_ nanoglass of 10 nm grain diameter at 8% and 16% total strain. Center: Atomic shear strain in a composite nanoglass of 10 nm grain diameter at 8% and 16% total strain. Right: Atomic shear strain in a Cu_36_Zr_64_ nanoglass of 10 nm grain diameter at 8% and 16% total strain. The symbols show the position of Cu- and Zr-rich grains, respectively. Reproduced with permission from [52].

Their yield stresses were significantly lower than the one of the corresponding bulk glass. This reduction of the yield stress seems to result from the lower nucleation barrier for shear transformation zones (STZ) at the nanoglass interfaces [53]. Since shear band propagation is driven by the local elastic energy, the local energy release is not sufficient to accelerate one of these local STZ so that it became a shear band. As a consequence, both nanoglasses deformed homogeneously (Figure 19). A comparable effect has already been reported for metallic glasses that were pre-deformed by cold rolling [48]. In fact, it may be described in a more quantitative way by the strain localization parameter proposed by Cheng et al. [37]. These results are in line with observations on pre-induced shear bands produced by indentation [37] or cold rolling [54] where structural disorder was found to be retained even after annealing. The effect of the chemical composition of the glassy regions on the plastic deformation of nanoglasses was studied by using a layered nanoglass composite consisting of Cu_64_Zr_36_ and Cu_36_Zr_64_ grains (Figure 20). Snapshots of the distribution of atomic shear strains are shown in Figure 20 for strains of 8% and 16%. Clearly, in all cases STZs are activated at the interfaces.

#### Biocompatibility of nanoglasses

Due to their high strength, large elastic limit and excellent corrosion resistance, metallic glasses are considered to be promising biomaterials. As one of the most widely used implantable metals, titanium and its alloys have attracted considerable scientific and technological interest. In fact, substantial efforts were devoted to the development of biocompatible Ti-based bulk metallic glasses (BMGs) [55–57].

However, without Ni, which is toxic to the human body, Ti-based alloys generally exhibit a lower glass-forming ability [58–60] than the other metal-based BMGs, for example, Zr-, Cu- or Fe-based alloys.

**Figure 21:**
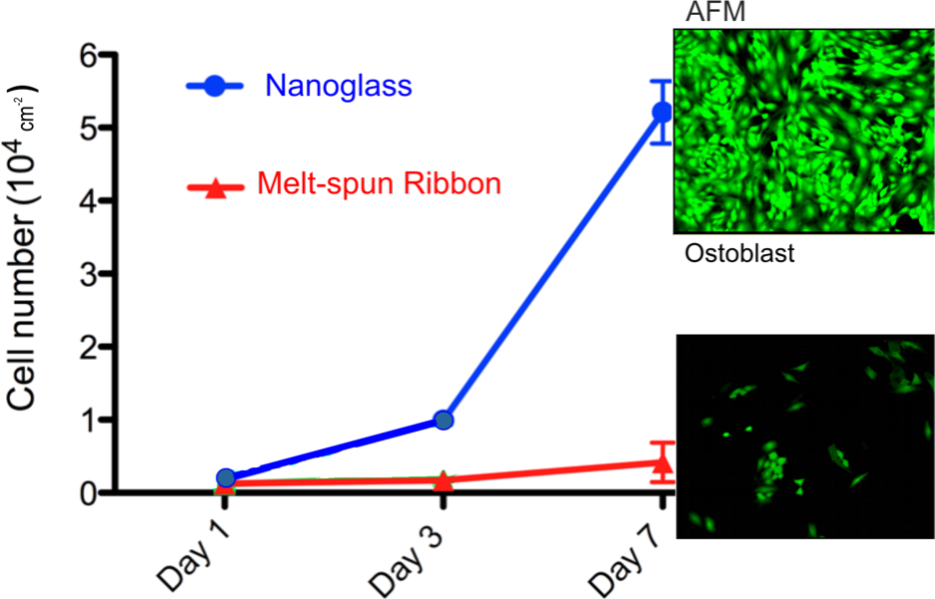
Cell proliferation at the surface of a melt-spun ribbon and at the surface of a nanoglass with the same chemical composition (Ti_34_Zr_14_Cu_22_Pd_30_). The micrographs on the right side display the density of the osteoblasts (green color) on the surfaces of both materials after a growth time of 7 days [61]. Reproduced by permission of the Royal Chemical Society.

On the other hand, it is known that the cellular response to materials is significantly influenced by the microstructure of the implanted materials, their surface roughness, their surface topography and their chemical compositions. In order to study [61] the effect of the nanoscale microstructure of nanoglasses on the bioactivity, hierarchically structured layers of Ti_34_Zr_14_Cu_22_Pd_30_ metallic nanoglass were created by magnetron sputtering. The cell proliferation on the surfaces of these materials was studied by seeding ten thousand osteoblasts on the free surface of the Ti_34_Zr_14_Cu_22_Pd_30_ metallic nanoglass, on the free surfaces of metallic glass ribbons (same chemical composition) with rough (MGR) and smooth surfaces (MGS), as well as on the free surface of pure Ti. As can be seen from Figure 21, the cell density on the surface of the Ti_34_Zr_14_Cu_22_Pd_30_ nanoglass was about fifteen times higher than that on the surface of the corresponding melt-spun ribbon. Moreover, it was about five-fold and about ten-times higher than the cell densities on surfaces of the MGR and MGS ribbons, respectively. This high level of cell proliferation does not seem to be caused primarily by the surface roughness of the Ti_34_Zr_14_Cu_22_Pd_30_ nanoglass. Both sides of the Ti_34_Zr_14_Cu_22_Pd_30_ glassy ribbons had a roughness that was comparable to the one of the Ti_34_Zr_14_Cu_22_Pd_30_ nanoglass. Despite the comparable roughness, the ribbons displayed a lower bioactivity than the nanoglass and the Ti control specimen.

The significance of nanometer-sized patterning of the surface of nanoglasses agrees with the results of recent studies [62–64] indicating that the spatial patterning of biochemical cues controls several cellular processes such as spreading, adhesion, migration and proliferation. In fact, these studies indicate that the lateral spacing of individual integrin receptor-ligand bonds determines the strength of cellular adhesion. For spacings larger than about 90 nm focal contact formation was found to be inhibited and detachment forces were significantly smaller than for spacings below 50 nm. This seems to be so because integrin clustering and adhesion-induced arginine-glycine-aspatic acid (RGD) ligands depend on the local order of the ligand arrangement on the substrate if the average ligand spacing is above 70 nm. Adhesion is “turned off“ by RGD patterning above 70 nm and “turned on” below this spacing.

#### Catalytic properties

Organosilanols are utilized to synthesize silicon-based polymer materials [65–66]. Moreover, organosilanols are also important as coupling partners in metal-catalyzed coupling reactions [67–70]. As a consequence, methods for removing silanes by an oxidation process of silanes with water are attractive from an environmental point of view. This oxidation process with water (Equation 1) should be catalyzed by a heterogeneous catalyst so that the coproduct of this oxidation process would be nonpolluting hydrogen gas [71–75]. The Au_52_Ag_5_Pd_2_Cu_25_Si_10_Al_6_ nanoglass was noted [7] to exhibit a high catalytic activity for the reaction (Equation 1).

[1]
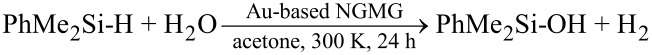


In fact, when dimethylphenylsilane was heated with H_2_O in the presence of Au_52_Ag_5_Pd_2_Cu_25_Si_10_Al_6_ nanoglass, the yield of the reaction (after 24 h at 20 °C) catalyzed by Au_52_Ag_5_Pd_2_Cu_25_Si_10_Al_6_ nanoglass was 93%, whereas only trace amounts of dimethylphenylsilanol were obtained, under the same conditions, with Au_52_Ag_5_Pd_2_Cu_25_Si_10_Al_6_ glassy ribbons having flat surfaces.

Silanols are known to form disiloxanes in the presence of even trace amounts of acid or base. However, in the reaction catalyzed by Au_52_Ag_5_Pd_2_Cu_25_Si_10_Al_6_ nanoglass, the formation of such a by-product did not occur. His conclusion was suggested by the fact that the by-products were not detected at all by gas-chromatography–mass-spectrometry. Moreover, the oxidation process seems to have occurred exclusively at the surface of the Au_52_Ag_5_Pd_2_Cu_25_Si_10_Al_6_ nanoglass catalyst. This conclusion was suggested by the results of leaching measurements.

### Multiphase nanoglasses

#### Production of multiphase nanoglasses

**Figure 22:**
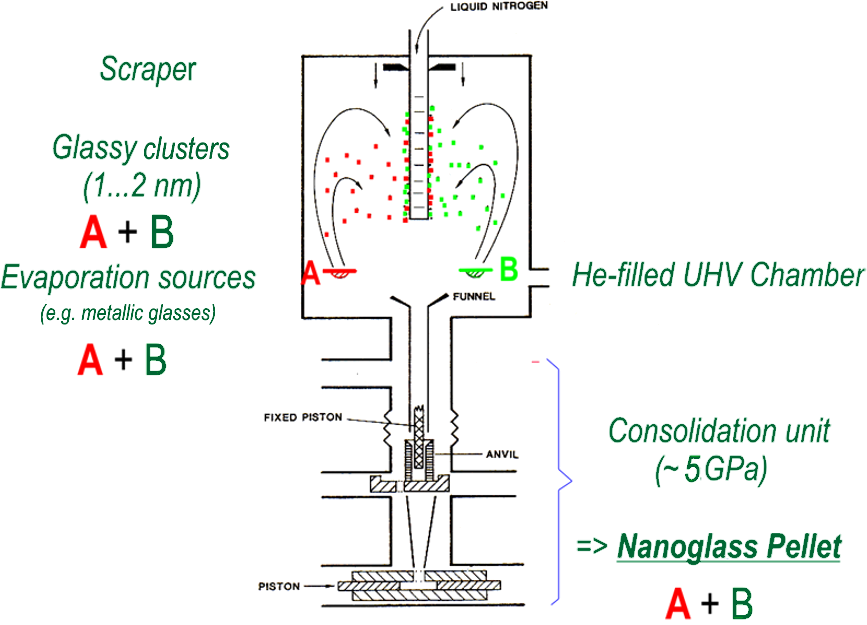
Production of multiphase nanoglasses by the consolidation of glassy clusters with different chemical compositions (A and B). The clusters are generated by evaporating both components simultaneously in an inert gas atmosphere and by collecting the two kinds of clusters at the surface of a cold finger [6,78]. Reproduced with permission from [6].

So far multiphase nanoglasses have been produced by inert-gas condensation (IGC) [6] and by phase separation on a nanometer scale [76–77]. The first multiphase glasses structured on a nanometer scale have been produced by inert-gas condensation and consisted of a mixture of nanometer-sized glassy regions of a FeSc metallic glass and of a Cu_70_Sc_30_ metallic glass [78]. The production was performed by arranging two evaporators in the IGC device as shown in Figure 22.

**Figure 23:**
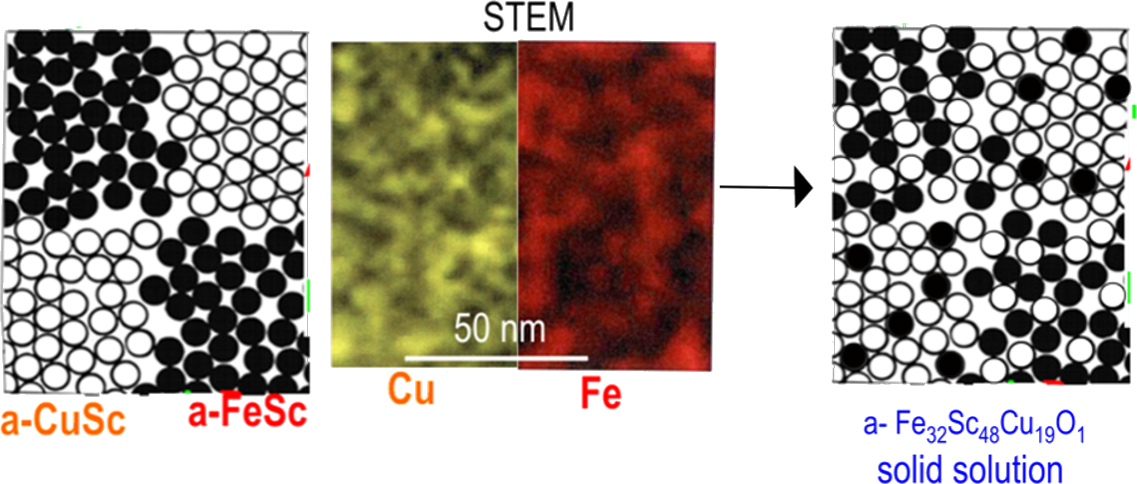
Structure of a two-phase nanoglass consisting of FeSc and Cu_70_Sc_30_ glassy clusters (Figure on the left side). The STEM micrographs in the center display the heterogeneous elemental distribution in the two-phase nanoglass after preparation [78]. The atomic structure of the glass after annealing is indicated by the figure on the right side. After annealing, interdiffusion of Fe, Sc and Cu atoms within the clusters results in a chemically homogeneous nanoglass with a composition of Fe_32_Sc_48_Cu_19_O_1_. This composition evidences that it is possible to generate a solution of Fe–Sc–Cu although these elements are practically immiscible in the crystalline state.

Observations by TEM and WAXS confirmed the expected microstructure of the multiphase nanoglass. In fact, the FeSc–Cu_70_Sc_30_ two-phase nanoglass turned out to be a random mixture of both kinds of glassy clusters that formed an amorphous solid solution upon annealing. This result appears remarkable because Fe and Cu are practically immiscible in the crystalline state at similar temperatures (Figure 23).

Although the microstructures of multiphase nanocrystalline materials and of multiphase nanoglasses appear similar, there is the following basic difference between the two kinds of nanomaterials. As is well known from the phase diagrams of numerous alloys, the mutual solubility of the components forming an alloy is, in most systems, in the molten state much higher than the mutual solubility of the same components in the crystalline state. Well-known examples for the different solubilities in the melt and in the crystalline state are the high solubilities of NaCl or sugar in water and the low solubility of NaCl or sugar in ice. As a consequence of these different solubilities, it is expected that in multiphase nanoglasses, one will be able to obtain glassy solid solutions of components that are immiscible in the crystalline state.

Multiphase glasses structured on a nanometer scale have been produced by phase separation and have been studied in several alloy systems, e.g., in Ag–Ni [79–80], Cu–Nb [81], Ag–Cu [82–84], Ag–Fe [56], Ag–Gd [57], Cu–Ta and Cu–W [58]. The nanostructured glasses produced by phase separation differ from the ones prepared by the IGC method primarily by the structure of the interfaces between adjacent regions of different chemical compositions and by the limitations in selecting the chemical compositions of the regions A and B (Figure 3h). In the case of multiphase nanoglasses produced by IGC, the chemical compositions of the components may be selected freely as long as it is possible to generate nanometer-sized glassy clusters.

#### Semicrystalline multiphase nanoglasses

**Figure 24:**
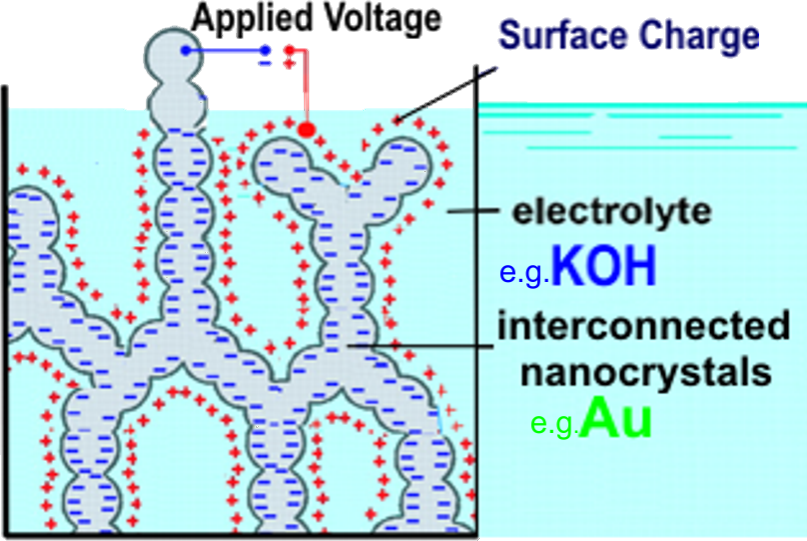
Generation of an electrically charged surface in a nanoporous metal (e.g., Au) if it is immersed into a suitable electrolyte (here KOH) and if a voltage is applied between the metal and the electrolyte so that a double-layer is formed at the surface of the nanoporous metal [85–87]. Reproduced with permission from [86].

In this class of multiphase nanoglasses, one phase is a nanometer-sized crystalline material, the other phase has an amorphous structure either in the form of a liquid, a glass or a gas. The underlying concept of these materials with liquid/glass/gas-filled ligaments is that the properties of the interfaces between the nanostructured crystalline and liquid/glassy/gassy components can be tuned by variation of the state variable in the surrounding medium, for instance, the electric or chemical potential of the electrolyte in the ligaments, the gas pressure or the chemical composition of the liquid [85]. As an example, Figure 24 illustrates the induced electric charge in the surface region of the metal due to an electrochemical double layer at the interface between the electrolyte and the nanoporous metallic material. By means of this induced electric charge, all properties of the nanoporous metal that depend on its electronic structure may be tuned.

As multiphase nanoglasses are beyond the scope of this review, we would like to refer the reader to some of the recent publications in that area [8,81,86–109].

## Conclusion

This paper started by considering the role of materials in the history of mankind. Hence it seems appropriate to close the paper by considering the conceivable historical implications of the development reported here.

In the past, the understanding and utilization of materials such as metals, semiconductors, ceramics, etc., resulted in specific periods in the development of mankind. In fact, the names of some of these periods were selected according to these materials such as the Iron Age, the Bronze Age etc. All of these periods are characterized by the fact that the properties of the new materials that became available by controlling their structure were utilized and permitted new technologies to be developed. Today, we seem to be in a comparable situation for materials with noncrystalline structures. In fact, nanoglasses seem to open the way to a new class of noncrystalline materials with controllable atomic and electronic structures and, hence, new properties (in comparison to glasses produced by quenching the melt). Hence, by analogy with the developments of the past, nanoglasses may permit the development of technologies that are not possible today by utilizing the new properties of nanoglasses.

A prerequisite for a development of this kind is, however, that one succeeds in developing economic methods for producing large quantities of nanoglasses with well-controlled microstructures.
